# Study of the variation of the 12-month prevalence of exposure to workplace bullying across national French working population subgroups

**DOI:** 10.1007/s00420-022-01916-x

**Published:** 2022-09-03

**Authors:** Isabelle Niedhammer, Elodie Pineau, Sandrine Bertrais

**Affiliations:** INSERM, Univ Angers, Univ Rennes, EHESP, IRSET (Institut de Recherche en Santé, Environnement et Travail)-UMR_S 1085, ESTER Team, Angers, France

**Keywords:** Workplace bullying, Violence, Occupational exposure, Working population

## Abstract

**Objectives:**

The studies are lacking on the variation of the prevalence of exposure to workplace bullying according to subgroups of national working populations. The objectives were to assess the 12-month prevalence of bullying in the national French working population, to describe the reported reasons for bullying, and to study its variation according to various employment variables.

**Methods:**

The study was based on the data of the 2013 national French working conditions survey. The study sample included 25,769 employees aged 15–65 working in the same job within the last 12 months. The 12-month prevalence of bullying was assessed using a 9-item questionnaire. Employment variables included: occupation, economic activity of the company, public/private sector, company size, permanent/temporary work contract, and full/part-time work. The analyses were performed using statistical methods for weighted survey data.

**Results:**

The 12-month prevalence of bullying was 26.7% and 28.7% for men and women, respectively. The most prevalent forms of bullying were criticisms, exclusion, and deprivation of right of expression. The leading reasons for being bullied were related to occupation, age, and gender. The prevalence of bullying was higher among the younger employees, the employees working in medium/large companies (including the public sector), and among employees working full time. Though significant, the variations according to occupations and economic activities of the company were small.

**Conclusion:**

Workplace bullying appeared as a widespread phenomenon in France. More attention should be given to young employees and the employees working in medium/large companies. Preventive measures should also target the whole working population comprehensively.

**Supplementary Information:**

The online version contains supplementary material available at 10.1007/s00420-022-01916-x.

## Introduction

Workplace bullying is a major occupational hazard in developed countries and has been found to be associated with various health outcomes especially mental disorders (Niedhammer et al. 2021; Verkuil et al. 2015), including depression-related outcomes (Theorell et al. 2015) and sleep problems (Nielsen et al. 2020).

Workplace bullying is difficult to define and various definitions have been suggested without clear consensus. According to Leymann (1996), workplace bullying “involves hostile and unethical communication, which is directed in a systematic way by one or a few individuals mainly towards one individual who is pushed in a helpless and defenceless position”. Questionnaires have been developed to assess the exposure to bullying and to cover various forms of bullying. A harmonized European study showed that there were differences in the prevalence of exposure to bullying between countries and that France had a particularly high prevalence of exposure (Niedhammer et al. 2022). The prevalence of exposure depends on the used questionnaire and on the studied time period of the prevalence. In France, for example, the point prevalence of bullying was found to be around 16% in the national working population in 2016 using 9 items related to 9 forms of bullying (Niedhammer et al. 2020) and 7.5% in the South–East of France in 2004 using Leymann’s questionnaire called LIPT assessing 45 forms of bullying (Niedhammer et al. 2007). The point prevalence measures the prevalence at a specified point in time and is expected to be low. Consequently, a longer period of time leads mechanically to a higher prevalence of exposure. Nevertheless, due to recall effect, it may be difficult to determine a proportionality between the period of time and the magnitude of the prevalence. In addition, it is not obvious to assess the impact of the number of items (forms of bullying) used in the questionnaire on the prevalence of exposure. The differences in measurement methods lead to a large variability in the prevalence of exposure observed in the literature, between studies, and even within the same country.

Information about the variation of the prevalence of exposure to workplace bullying across subgroups of the working population may be useful to determine highly exposed groups and orient preventive strategies. In a previous publication (Niedhammer et al. 2007), we showed that the point prevalence displayed some variations according to occupation and economic activity of the company in France. Some or few variations according to these variables were also observed in other countries (Alterman et al. 2013; Hoel et al. 2001; Hubert and van Veldhoven 2001; Lange et al. 2019; Leymann 1996; Ortega et al. 2009; Tsuno et al. 2015). Nevertheless, no clear pattern has been found so far. In the literature, there was a lack of national representative studies on the variation of the prevalence of exposure to bullying according to subgroups of the working population. Indeed, most previous studies focused on samples from specific occupational groups or sectors/industries. In addition, the existing studies often suffered from a lack of statistical power and of formal statistical testing. Furthermore, if overall difference tests were performed, most of the studies failed to provide 95% Confidence Intervals (CIs) of the estimates of the prevalence across subgroups, leading to potential erroneous conclusions.

Our objective was to assess the 12-month prevalence of exposure to workplace bullying, to describe the reported reasons for this exposure, and to explore the variation of this prevalence according to various employment variables in the national French working population of employees.

## Methods

The study was based on the data of the 2013 national French Working Conditions survey, conducted by the DARES of the French ministry of labour, that included a nationally representative sample of 29,556 employees aged 15–65. The survey had a two-stage random sampling design that selected, first, households and, then, workers, if more than one worker within the household. Data collection was done using a questionnaire asked by an interviewer and a self-administered questionnaire for more sensitive questions.

### Workplace bullying

In the national French Working Conditions survey, workplace bullying was assessed in the self-administered questionnaire using 9 items derived and inspired from the LIPT questionnaire including 45 items (Leymann 1996), which was translated and validated in French previously (Niedhammer et al. 2006). The choice of 9 items was done to reduce the length of the LIPT questionnaire and content redundancies between items of the LIPT questionnaire. For example, item #1 (Ignore(s) you, behave(s) as if you were not there) was retained instead of 3 items of the LIPT questionnaire with close content (Leymann 1996; Niedhammer et al. 2006). Similarly, item #2 (Prevent(s) you from expressing yourself) replaced 3 items of the LIPT questionnaire, etc. These 9 items were already used in another national survey in France, the SUMER survey (Niedhammer et al. 2020). The 9 items are related to 9 forms of bullying that people may have experienced within the last 12 months. The 9 items are presented in Appendix. The study of the 12-month prevalence of exposure allowed us to have a higher number of exposed people and a higher statistical power than the point prevalence that was used in our previous study using the national SUMER survey (Niedhammer et al. 2020). Another item was also used and assessed whether the perpetrator was employee(s) within the company. Therefore, workplace bullying was defined by the exposure within the last 12 months to at least one of the 9 forms of bullying whose perpetrator was someone within the company. As the prevalence of exposure was related to the last 12 months, we restricted the sample to the employees who were working in the same job within the last 12 months.

For the exposed employees, 8 items were asked about the reason(s) the employees thought they were bullied: (1) gender, (2) health status or disability, (3) skin colour, (4) country of origin or nationality, (5) ways of dressing, (6) age, (7) sexual orientation, and (8) occupation.

### Employment variables

In addition to gender and age, we studied various employment variables in association with workplace bullying:

- 3 variables for occupation coded using the French classification (INSEE 2003). These 3 variables were related to different levels of the classification and included 4, 14, and 25 groups, respectively.
- 3 variables for economic activity of the company coded using the French classification (INSEE 2008). These 3 variables were related to different levels of the classification and included 4, 17, and 38 groups, respectively.
- public/private sector of the company
- company size (total number of employees): small (< 50 employees), medium (50–499 employees), and large (500 or more employees)
- temporary/permanent work contract
- full/part-time work.

### Statistical analysis

All the statistical analyses were performed using weighted data to take non-response and marginal calibration into account. We made a description of the study sample according to the studied variables and tested the differences between genders using the Rao–Scott Chi-2 test. First, the associations between employment variables and workplace bullying were tested using the Rao–Scott Chi-2 test among the whole study sample. Second, Poisson regression models with robust variance estimation were performed to test the interactions between gender and employment variables in association with bullying. In case of significant gender-related interactions, the results were presented for men and women separately. Forest plots were used to present the 12-month weighted prevalence of exposure to bullying and 95% CIs according to subgroups. Sensitivity analyses included three additional analyses: (1) study of the associations of each employment variable with bullying with adjustment for age, (2) study of the associations of all employment variables simultaneously with bullying using robust Poisson regression models, and (3) study of all employment variables in association with bullying using forward stepwise robust Poisson regression models (*p* value < 0.05 as criterion for entry into the model). All the analyses were done using R software.

## Results

### Description of the study sample

Among the 28,121 employees aged 15–65 who responded to the self-administered questionnaire (response rate: 95.1%), 25,769 were working in the same job within the last 12 months and constituted our study sample.

The description of the study sample according to gender, age, and employment variables is presented in Supplementary Table S1. Gender differences in the distribution of all variables were observed. Women were more likely to be older than men. Women were more likely to work as associate professionals/technicians and clerks/service workers than men, and men were more likely to work as professionals/managers and blue collar workers than women. Women were more likely to work in the services than men, and men in agriculture, construction, and manufacturing than women. Women were more likely to work in the public sector and small companies, have temporary work contract and work part-time than men.

### 12-Month prevalence of workplace bullying

The description of the 12-month prevalence of exposure to the 9 bullying forms is presented in Table 1. The highest prevalence was found for item #4 (Unfairly criticize(s) your work) and item #1 (Ignore(s) you, behave(s) as if you were not there), and the lowest prevalence for item #9 (Make(s) sexual proposals to you insistently). Women were more likely to be exposed to item #1 (Ignore(s) you, behave(s) as if you were not there), item #2 (Prevent(s) you from expressing yourself), item #3 (Ridicule(s) you in public), and item #9 (Make(s) sexual proposals to you insistently) than men, whereas men were more likely to be exposed to item #5 (Burden(s) you with useless or degrading tasks) than women. There was no gender difference in the distribution of the number of bullying forms: 9.8% of the employees were exposed to one form of bullying, 6.5% to two forms, and 11.4% to three forms or more. The 12-month weighted prevalence of bullying, as defined by the exposure to at least one form of bullying from employee(s) within the company, was found to be 27.7% (95% CI 26.8–28.7) among the whole study sample, with a significant but small difference between men (26.7%, 95% CI 25.4–28.1) and women (28.7%, 95% CI 27.4–30.1).

**Table 1:** Description of the 12-month weighted prevalence of exposure to the 9 forms of workplace bullying among the study sample and among men and women separately

	All (*N* = 25,636) *n* (w%)	Men (*N* = 10,926) *n* (w%)	Women (*N* = 14,710) *n* (w%)	*p* value
1. Ignore(s) you, behave(s) as if you were not there	4518 (16.3%)	1758 (15.5%)	2760 (17.2%)	0.034
2. Prevent(s) you from expressing yourself	2763 (10.2%)	999 (8.9%)	1764 (11.4%)	< 0.001
3. Ridicule(s) you in public	1695 (6.4%)	650 (5.8%)	1045 (6.9%)	0.044
4. Unfairly criticize(s) your work	4633 (18.0%)	1952 (17.8%)	2681 (18.2%)	0.668
5. Burden(s) you with useless or degrading tasks	1786 (6.8%)	829 (7.6%)	957 (6.0%)	0.003
6. Sabotage(s) your work, prevent(s) you from working properly	1824 (6.8%)	746 (7.0%)	1078 (6.6%)	0.394
7. Insinuate(s) that you are mentally disturbed	670 (2.5%)	296 (2.5%)	374 (2.5%)	0.988
8. Say(s) obscene or degrading things to you	911 (3.8%)	394 (3.9%)	517 (3.7%)	0.681
9. Make(s) sexual proposals to you insistently	207 (0.7%)	47 (0.4%)	160 (0.9%)	0.002
Number of bullying forms				0.215
0	18,262 (72.3%)	7940 (73.3%)	10,322 (71.3%)	
1	2626 (9.8%)	1083 (9.5%)	1543 (10.1%)	
2	1731 (6.5%)	697 (6.3%)	1034 (6.8%)	
3 or more	3017 (11.4%)	1206 (10.9%)	1811 (11.8%)	
12-Month prevalence of workplace bullying^a^	7374 (27.7%)	2986 (26.7%)	4388 (28.7%)	0.036

*n* (w%): unweighted number (weighted %)

*p* value for the comparison between genders (Rao–Scott Chi-2 test)

^a^Workplace bullying was defined by exposure to at least one form of bullying (among the 9 items) from employee(s) within the company

### Reported reasons for bullying

A total of 7374 employees were classified as exposed to workplace bullying within the last 12 months. Among them, the most frequently reported reason for being bullied was occupation (23.7%), and women were more likely to report this reason than men (Table 2). The second most frequently reported reason was age (17.1%), which was reported more frequently by women than by men. Additional results showed that the age-related reason was reported more frequently among the youngest (34.2%) and oldest employees (20.1%). The third most frequently reported reason was gender (13.7%), with a massive difference between men (3.9%) and women (22.7%). Four reasons for being bullied (health status/disability, skin colour, origin/nationality, and ways of dressing) displayed similar percentages, around 4–7%. There was no gender difference for the reason related to health status/disability, whereas there were small gender differences for the three other reasons. The least frequently reported reason was sexual orientation (2%), which was reported more frequently among men than among women. There was a gender difference in the distribution of the number of reported reasons, as women were more likely to report a higher number of reasons than men. It may also be informative to point out that half of the exposed employees did not provide any reason for being bullied, and this was the case more for men than for women (57.5% versus 45.5%), suggesting that there may be other reasons or that employees did not know the reasons why they were bullied.

**Table 2:** Reported reason(s) for being bullied among all the exposed employees, and among the exposed men and women separately

	All *n* (w%)	Men *n* (w%)	Women *n* (w%)	*p* value
Number of exposed employees	7374	2986	4388	
Bullied because of… (*multiple answers possible*)				
Gender	1000 (13.7%)	123 (3.9%)	877 (22.7%)	< 0.001
Health status or disability	406 (5.5%)	163 (5.6%)	243 (5.5%)	0.897
Skin colour	353 (4.2%)	177 (5.4%)	176 (3.1%)	< 0.001
Origin or nationality	493 (7.0%)	256 (8.7%)	237 (5.5%)	0.001
Way of dressing	403 (5.5%)	144 (4.3%)	259 (6.7%)	0.008
Age	1119 (17.1%)	391 (14.3%)	728 (19.7%)	0.002
Sexual orientation	117 (2.0%)	67 (2.9%)	50 (1.2%)	0.002
Occupation	1808 (23.7%)	681 (21.0%)	1,127 (26.1%)	0.004
Number of reported reasons				< 0.001
0	3,756 (51.2%)	1,685 (57.5%)	2,071 (45.5%)	
1	2,312 (30.6%)	887 (28.8%)	1,425 (32.3%)	
2	837 (11.1%)	261 (8.4%)	576 (13.7%)	
3 or more	469 (7.0%)	153 (5.4%)	316 (8.5%)	

*n* (w%): unweighted number (weighted %)

*p* value for the comparison between genders (Rao–Scott Chi-2 test)

### Variation of the 12-month prevalence of bullying according to subgroups

**Fig. 1.**
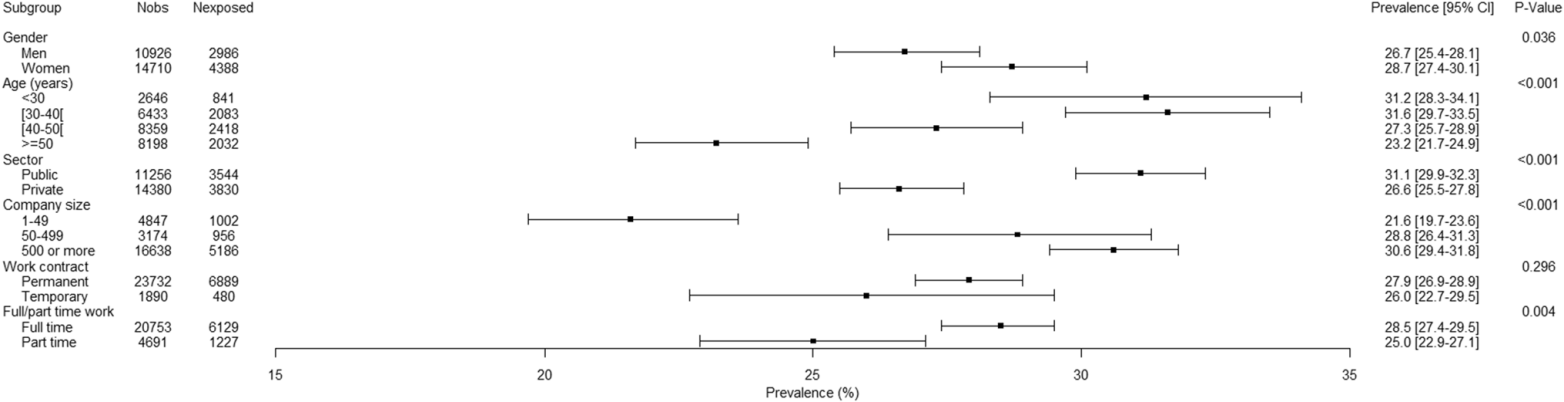
12-Month weighted prevalence of exposure to workplace bullying according to gender, age, public/private sector, company size, work contract, and full/part-time work among the study sample

There were no gender-related interaction with age, public/private sector, company size, work contract, and full/part-time work in association with bullying. Consequently, the associations of these variables with bullying were presented among the whole study sample (Fig. 1). The oldest employees (aged 50 or more) had a lower prevalence of bullying, and the youngest (aged less than 40) had a higher prevalence. No association was observed between work contract and bullying. The employees working full time, working in the public sector and working in medium/large companies had a higher prevalence of bullying. As the public sector was part of large companies (500 employees or more), we also studied the association between company size and bullying among the subsample of the employees working in the private sector. Among this subsample, this association was significant (*p* < 0.001) and the prevalence of exposure to bullying was 21.6%, 28.8%, and 30.2%, respectively, in small, medium, and large companies.

**Fig. 2.**
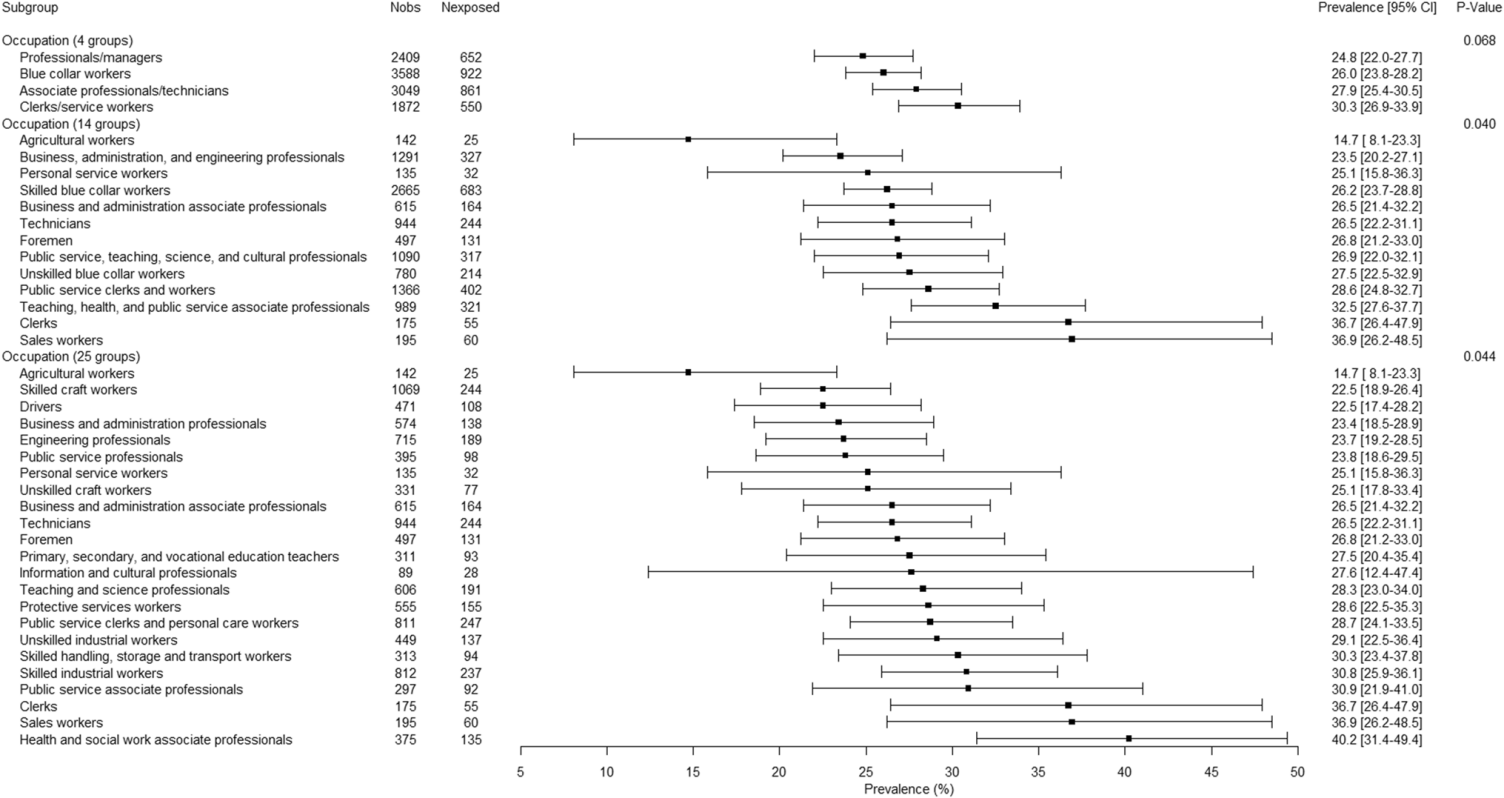
12-Month weighted prevalence of exposure to workplace bullying according to occupation among men (Two occupational groups were not presented in the Figure because of very low sample size: Professionals working partially as self-employed and Clergy. Occupational groups are presented in increasing order of exposure prevalence.)

**Fig. 3.**
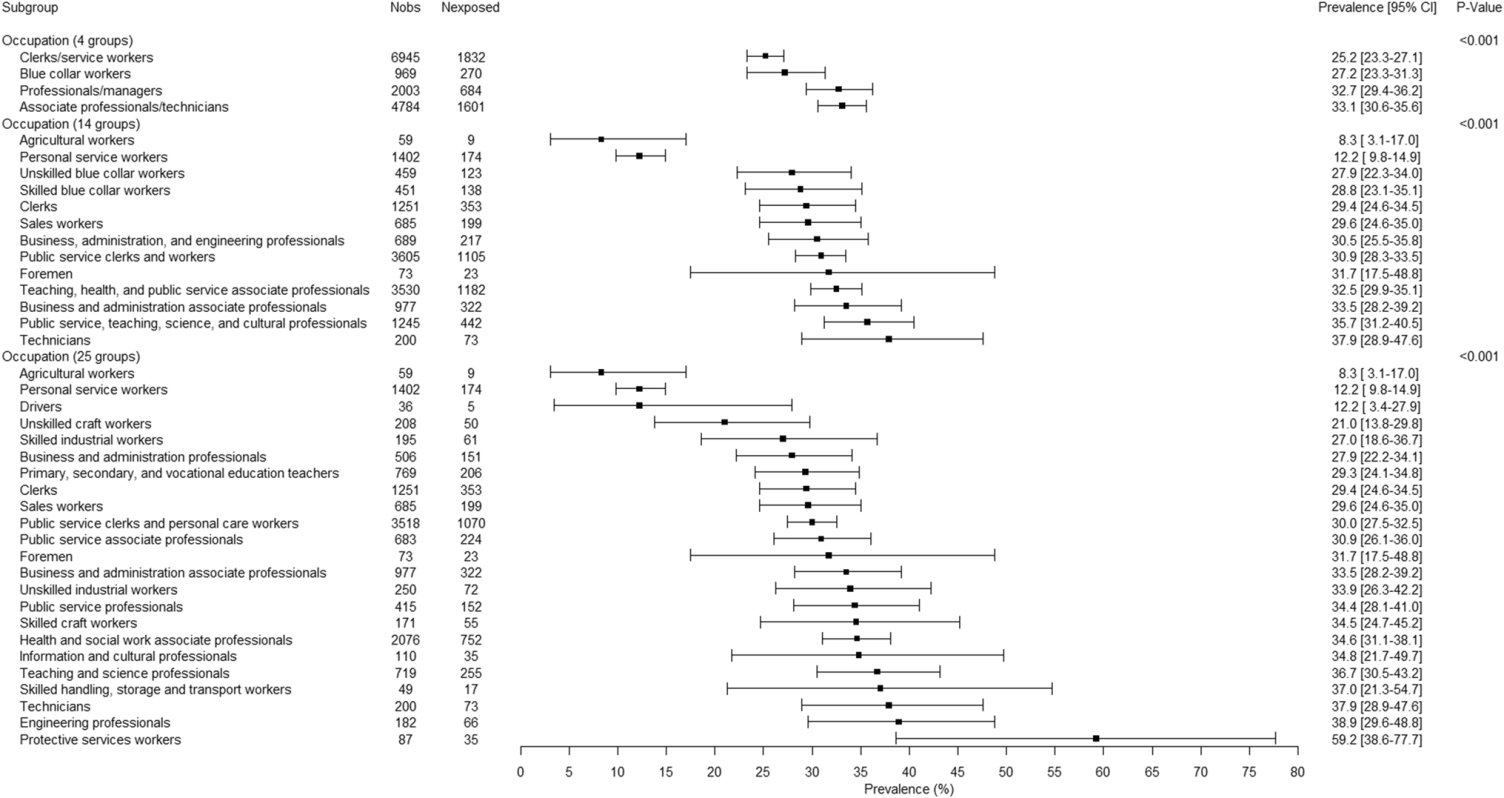
12-Month weighted prevalence of exposure to workplace bullying according to occupation among women (Two occupational groups were not presented in the Figure because of very low sample size: Professionals working partially as self-employed and Clergy. Occupational groups are presented in increasing order of exposure prevalence.)

**Fig. 4.**
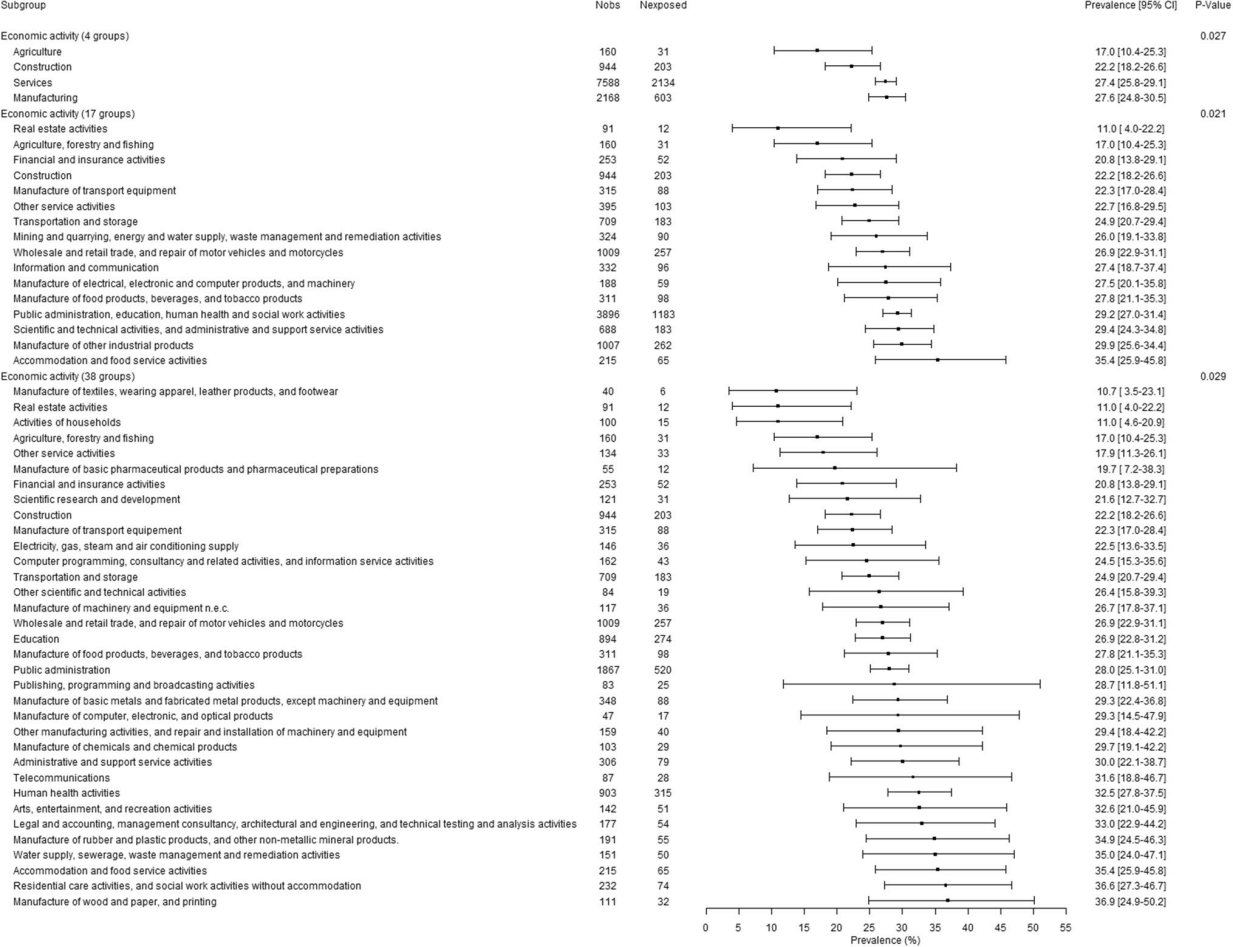
12-Month weighted prevalence of exposure to workplace bullying according to economic activity of the company among men (Four economic activities were not presented in the Figure because of very low sample size: Mining and quarrying, Manufacture of coke and refined petroleum products, Manufacture of electrical equipment, and Activities of extraterritorial organisations and bodies. Economic activities are presented in increasing order of exposure prevalence.)

**Fig. 5.**
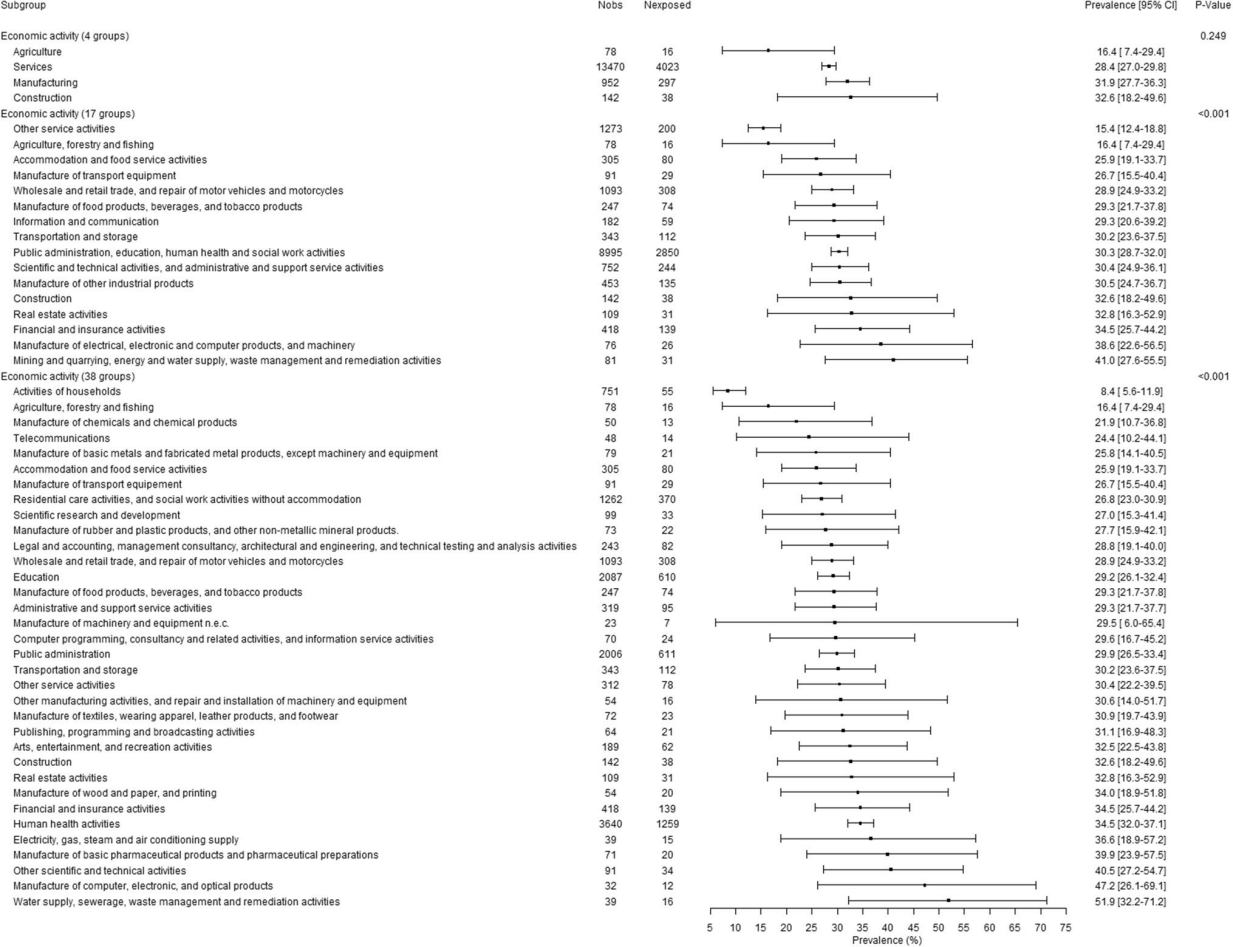
12-Month weighted prevalence of exposure to workplace bullying according to economic activity of the company among women (Four economic activities were not presented in the Figure because of very low sample size: Mining and quarrying, Manufacture of coke and refined petroleum products, Manufacture of electrical equipment, and Activities of extraterritorial organisations and bodies. Economic activities are presented in increasing order of exposure prevalence.)

There were significant gender-related interactions in association with bullying for occupation (p < 0.01) and economic activity of the company (p < 0.05 except for economic activity with 4 groups). The presentation of the results was therefore done for men and women separately in Figs. 2, 3, 4 and 5. Although the association between occupation (whatever the level of the used occupation classification) and bullying was significant among men and among women, substantial overlaps in the 95% CIs were found (Figs. 2 and 3). Agricultural workers among both genders and personal service workers among women displayed lower prevalences of exposure to bullying. Although overlaps were observed in the 95% CIs and caution was needed in the interpretation of the results, the occupations found with the highest prevalence of exposure to bullying among men and among women tended to be occupations dominated by the opposite gender: for men, clerks, sales workers, and health and social work associated professionals that included 87.7%, 76.8%, and 81.7% of women respectively, and for women protective services workers that included 88.8% of men. Similarly to the results related to occupation, although significant associations were found between economic activity of the company and bullying, the differences between groups appeared inconclusive due to the overlap between 95% CIs (Figs. 4 and 5).

### Sensitivity analyses

Sensitivity analyses provided similar results. (1) After adjustment for age, the results of the associations of each employment variable with bullying were unchanged (Supplementary Figures S1–S2). (2) The study of all employment variables simultaneously in association with bullying did not change the results substantially (Supplementary Tables S2–S3). However, the association between the public sector and bullying was no longer significant. This was explained by the adjustment for company size, as the public sector was part of large companies. In other terms, we found that the prevalence of bullying increased with company size in the private sector, and there was no difference between private large companies and the public sector. (3) The results of the forward stepwise regression models (Supplementary Tables S4–S5) showed that age and company size were the selected variables in association with bullying among men. Among women, the selected variables were: occupation, age, and according to the model company size and full/part-time work or economic activity of the company.

## Discussion

### Main results

The 12-month prevalence of exposure to workplace bullying appeared elevated in the national French working population of employees. More than a quarter of this population was exposed. The most frequent forms of bullying were criticisms, exclusion, and deprivation of right of expression. The main reported reasons for being bullied were related to occupation, age, and gender, with major gender differences. However, more than half of the exposed employees did not report any reason, suggesting that there might be other reasons and/or that the reasons were unknown. Women had a slightly higher prevalence of exposure to bullying than men. The association between age and bullying showed that younger employees had a higher prevalence of bullying. The prevalence of bullying was higher among the employees working in medium/large companies (including the public sector), and among employees working full time. However, regarding the associations of occupations and economic activities of the company with bullying, the overlaps between confidence intervals impeded firm conclusions.

### Comparison with the literature

As the prevalence of exposure to bullying may be highly dependent on cultural factors, and as France was found to be the European country with the highest prevalence of bullying (Niedhammer et al. 2022), the comparison of the magnitude of the prevalence at national level may be difficult with other international studies. Our results on the magnitude of the 12-month prevalence of exposure among the national French working population were consistent with the previous results observed in France, underlying a high prevalence of exposure. Using the LIPT questionnaire with 45 items, i.e., a questionnaire exploring a higher number of forms of bullying, a 12-month prevalence of 38.3% among men and 41.2% among women was found in the working population in the South-East of France in 2004 (Niedhammer et al. 2006). The magnitude of the prevalence of the 9 studied forms of bullying was also in line with these previous results, with high prevalence of exposure to criticisms, exclusion, and deprivation of right of expression and low prevalence of exposure to sexual proposals.

The comparison of the variation of the prevalence of exposure to bullying according to subgroups of the working population may be difficult for lack of national studies and differences in measurement methods between studies. We found a significant but small difference in the prevalence of exposure between men and women, in line with previous French results showing either a small higher prevalence of exposure among women (Niedhammer et al. 2006) or no gender difference (Niedhammer et al. 2020). Our results echoed the international literature. Indeed, according to the review by Feijo et al. (2019), 13 studies reported that women were more likely to be bullied than men, whereas 11 studies showed no association between gender and bullying. Only 2 studies reported that men were more likely to be bullied than women. Our study underlined that older employees had at lower prevalence of bullying and younger employees a higher prevalence, in agreement with the international literature. According to the review by Feijo et al. (2019), 8 studies showed that younger workers (aged less 45) were more likely to be bullied, 9 studies found no association between age and bullying, and only 1 study reported that older workers were more likely to be bullied.

We found that there were significant associations between the studied employment variables and bullying. The most striking result was the following: the employees working in medium/large companies had a higher prevalence of bullying. The results from the literature on company size were inconclusive (González Trijueque and Graña Gómez 2010; Tsuno et al. 2015). In our study, the association between the public sector and bullying disappeared after adjustment for company size. In fact, there was an association between company size and bullying in the private sector, but no difference between private large companies and the public sector. Rare previous studies found a higher prevalence of bullying in the public sector (Alterman et al. 2013; González Trijueque and Graña Gómez 2010), but did not examine a potential confounding role of company size.

Our results suggested that there may be only small differences for the other studied employment variables. No association was observed in our study between work contract (temporary versus permanent) and bullying, which is consistent with the review by Feijo et al. (2019). Although the association between occupation and bullying was found to be significant in our study, the 95% CIs displayed strong overlaps and made conclusions difficult. According to the review by Feijo et al. (2019), the patterns were unclear on the differences in the prevalence of exposure to bullying according to occupational groups. Studies exploring national samples or samples including various occupational groups reported either no differences (Leymann 1996; Tsuno et al. 2015) or few differences (Alterman et al. 2013; Hoel et al. 2001; Lange et al. 2019; Ortega et al. 2009) between occupational groups. The study by Alterman et al. (2013) reported a few differences in hostile work environment including bullying between occupations in the US due to overlaps between 95% CIs. The study by Hoel et al. (2001) reported a few differences between workers, supervisors, middle, and senior managers in Great Britain. The study by Lange et al. (2019) found that the prevalence of bullying increased with lower socio-economic status, i.e., from academics/managers, semi-professionals, skilled workers, to unskilled workers in Germany. The study by Ortega et al. (2009) showed significant differences between 4 occupational groups in Denmark: unskilled workers had the highest prevalence of exposure to bullying and managers/supervisors the lowest prevalence. However, the association between more detailed occupational groups and bullying was found to be non-significant in the study by Ortega et al. (2009). Our previous study in the South–East of France (Niedhammer et al. 2007) also provided a few differences between occupational groups among men and no differences among women. In the present study, the groups that were found to be different from the others (i.e., presenting no or very low overlap in terms of 95% CIs) seemed to be occupational groups in which the employees were more likely to work alone such as personal service workers providing direct services to individuals, or agricultural workers, and consequently who were less likely to be exposed to bullying from another employee within the company. In fact, these groups would not be ‘population-at-risk’ following epidemiology principles, and this may explain the results. In the same way, the association between full-time work and bullying might be explained by the fact that full-time employees would be ‘population-at-risk’ for a longer period of time than part-time employees.

The results for the association between economic activity of the company and bullying may also be considered as inconclusive in our study. The overlap between 95% CIs was high and made the interpretation of the results difficult. Consequently, our conclusions may be that our study did not detect clear differences between economic activities. Previous studies exploring this association were seldom in the literature. The study by Alterman et al. (2013) found a few differences in hostile work environment between industries in the US due to overlaps in CIs. A study reported differences between 11 sectors in The Netherlands, based on an overall statistical testing, but no 95% CIs were provided (Hubert and van Veldhoven 2001). The study by Ortega et al. (2009) also found no differences between industrial groups in Denmark. Another study showed no difference between the three sectors (primary, secondary, and tertiary) in Japan (Tsuno et al. 2015). In our previous study in the South–East of France (Niedhammer et al. 2007), we found that employees working in the services had a higher prevalence of bullying than those in the other sectors among men. There was, however, no differences in the prevalence of bullying according to economic activity among women.

### Limitations and strengths of the study

A number of limitations should be noticed. No validated questionnaire was used to assess bullying, although the 9 items were already used in a previous national survey in France (Niedhammer et al. 2020). These 9 items were chosen and derived from the LIPT questionnaire (Leymann 1996) and its French version (Niedhammer et al. 2006), that includes 45 items; consequently, some items might be lacking to measure bullying. This might have led to a potential underestimation of the 12-month prevalence of exposure in our study. This might also have impacted the observed gender differences. However, most of the 45 items of the LIPT questionnaire did not display any difference between genders in France in a previous study (Niedhammer et al. 2006), reinforcing our present results suggesting little difference in bullying between genders. There was no assessment of duration and frequency of bullying, contrary to recommendations by numerous authors such as Leymann (1996) who defined the exposure to bullying by both a frequency of at least once a week and a duration of at least 6 months. This may have led to an overestimation of the prevalence of bullying in our study. We were not able to study the perpetrator precisely, i.e., whether employees were bullied by superiors, colleagues, or subordinates within the company (Lange et al. 2019). Nevertheless, we retained information about the perpetrator who was an employee (or employees) within the company, which was not the case previously (Niedhammer et al. 2020). We had also no information about the number and gender of perpetrators. We restricted the study sample to the employees who were working in the same job within the last 12 months, making the studied time period similar for the employment variables and bullying. Consequently, a number of employees who might have changed job because of bullying within the last 12 months might have been excluded from the study. This may have led to a potential underestimation of the 12-month prevalence of bullying. However, this underestimation is likely to be low as only 8.4% of the employees changed job within the last 12 months. There was no open-ended response option in the survey to collect other reasons for bullying that were not covered in the proposed list. This explains why we were unable to provide clearly identified reasons for bullying for around half of the study sample. This point deserves more attention in the future. Our study objective was to study the associations between the main employment variables and bullying. Consequently, our study did not claim to cover all variables associated with bullying.

Strengths of the study should be underlined. Our study was based on a national representative survey of the French working population of employees with a large sample size and high response rate (95.1%). We used weights to take non-response and marginal calibration into account and provided estimates that could be extrapolated to the whole national working population. We tested gender differences and gender-related interactions. Bullying was assessed using a 9-item questionnaire that was used previously in the national SUMER survey (Niedhammer et al. 2020). To this questionnaire was added one item on the perpetrator to be sure that the perpetrator was someone within the company, in accordance with the classical definition of bullying. Information was available on the reason(s) why the employees considered themselves as being bullied, which may be informative on a preventive point of view. The questions related to bullying were asked in the self-administered questionnaire, as these questions may be considered sensitive. This may have reduced reporting bias. The prevalence of exposure relied on a 12-month period making the study more powerful than previous studies using point prevalence or 6-month prevalence. Our statistical analyses were based on both statistical tests and 95% CIs’ calculation, allowing us to be more cautious in our interpretation of the differences between subgroups. We took employment seniority into account, to make similar the 12-month period for the assessment of both employment variables and bullying, to avoid misclassification due to bullying that might have been related to a previous employment. Our study covered various employment variables and included various levels of the classifications for occupation and economic activity of the company.

### Implications of the findings

Three implications of our results may be underlined. First, the associations between employment variables and workplace bullying, though significant, did not display clear patterns, except for the results of company size. Our results suggested that employment variables such as work contract, occupation, and economic activity of the company may not be major determinants of bullying. Second, our results may suggest that workplace bullying cannot reasonably be assessed using a job-exposure matrix, a tool that is commonly used in epidemiological studies, that aims at assessing exposure at job level (job title may be occupation, combined or not with economic activity of the company) and not at individual level (Niedhammer et al. 2018), and that may particularly be useful when no individual exposure assessment is available. In other words, our findings suggest that there would be no alternative to individual assessment to assess workplace bullying and that individual assessment would be indispensable. Third, our results would imply that prevention should be oriented towards the whole working population as a comprehensive measure to prevent workplace bullying. Our findings also showed that young employees and employees working in medium/large companies should be given more attention. Although our results should be interpreted with caution, our study suggested that the highest prevalence of exposure to bullying would be in opposite gender-dominated occupations. This point may also deserve more research and attention.

### Conclusion

Our study showed that there were some differences in the 12-month prevalence of exposure to bullying according to employment variables. The most marked results showed that the employees working in medium/large companies (including the public sector) had a higher prevalence of bullying. This should be explored more deeply to better understand and prevent bullying in medium/large companies (including the public sector). Another result which may be considered obvious was that employees working alone (away from other employees within the company) or working part-time were not or proportionally less ‘population-at-risk’ and consequently were less likely to be exposed to bullying. Regarding the associations of occupation and economic activity of the company with bullying, as suggested previously by Ortega et al. (2009), “any one could be at risk of being subjected to bullying regardless the type of job or industry group”. Our findings confirmed that workplace bullying may be widespread across all employment subgroups. Organizational and psychosocial work factors may play a role in bullying (Feijo et al. 2019). Individual and personality factors (for both the victim and perpetrator) and cultural factors including a lack of training and management towards bullying prevention may also be crucial. This suggests that more training and information may be useful to promote good practices, and prevent and detect bullying behaviours at the workplace. Such preventive measures should be oriented comprehensively towards the working population.

### Electronic supplementary material

Below is the link to the electronic supplementary material.

Supplementary file1 (DOCX 236 KB)

## Data Availability

The dataset used and analysed during the current study is available from the corresponding author on reasonable request.

## Appendix

The 9 items of workplace bullying used in the 2013 national French Working Conditions survey.

Within the past 12 months, have you experienced the following difficult situations at work? One or more people systematically behave(s) with you as follows:

1. Ignore(s) you, behave(s) as if you were not there
2. Prevent(s) you from expressing yourself
3. Ridicule(s) you in public
4. Unfairly criticize(s) your work
5. Burden(s) you with useless or degrading tasks
6. Sabotage(s) your work, prevent(s) you from working properly
7. Insinuate(s) that you are mentally disturbed
8. Say(s) obscene or degrading things to you
9. Make(s) sexual proposals to you insistently.
