# Supplementary material for: Study of the variation of the 12-month prevalence of exposure to workplace bullying across national French working population subgroups
Source: Int Arch Occup Environ Health. 2022 Sep 3;96(2):213–24. doi: 10.1007/s00420-022-01916-x (PMC9905178; doi:10.1007/s00420-022-01916-x)

Supplementary Table S1. Distribution of age and employment variables among the study sample and among men and women separately

|  | All (N=25,636)  n (w%) | Men (N=10,926)  n (w%) | Women (N=14,710)  n (w%) | P-value |
| --- | --- | --- | --- | --- |
| **Age (years)** |  |  |  | 0.004 |
| <30 | 2,646 (14.7%) | 1,186 (15.3%) | 1,460 (14.1%) |  |
| [30-40[ | 6,433 (25.1%) | 2,803 (26.5%) | 3,630 (23.8%) |  |
| [40-50[ | 8,359 (30.5%) | 3,545 (29.8%) | 4,814 (31.2%) |  |
| >=50 | 8,198 (29.7%) | 3,392 (28.4%) | 4,806 (30.9%) |  |
| **Occupation (4 groups)** |  |  |  | <0.001 |
| Professionals/managers | 4,412 (19.9%) | 2,409 (23.9%) | 2,003 (15.9%) |  |
| Associate professionals/technicians | 7,833 (26.5%) | 3,049 (25.5%) | 4,784 (27.5%) |  |
| Clerks/service workers | 8,817 (30.7%) | 1,872 (13.7%) | 6,945 (47.4%) |  |
| Blue collar workers | 4,557 (23.0%) | 3,588 (36.9%) | 969 (9.2%) |  |
| **Occupation (14 groups)** |  |  |  | <0.001 |
| Professionals working partially as self-employed | 93 (0.3%) | 26 (0.1%) | 67 (0.4%) |  |
| Public service, teaching, science, and cultural professionals | 2,335 (8.0%) | 1,090 (8.2%) | 1,245 (7.9%) |  |
| Business, administration, and engineering professionals | 1,980 (11.6%) | 1,291 (15.6%) | 689 (7.7%) |  |
| Teaching, health, and public service associate professionals | 4,519 (10.8%) | 989 (5.8%) | 3,530 (15.7%) |  |
| Business and administration associate professionals | 1,592 (8.0%) | 615 (6.3%) | 977 (9.6%) |  |
| Technicians | 1,144 (5.1%) | 944 (8.9%) | 200 (1.4%) |  |
| Foremen | 570 (2.6%) | 497 (4.5%) | 73 (0.7%) |  |
| Public service clerks and workers | 4,971 (12.2%) | 1,366 (8.3%) | 3,605 (16.2%) |  |
| Clerks | 1,426 (6.9%) | 175 (1.7%) | 1,251 (12.1%) |  |
| Sales workers | 880 (4.1%) | 195 (1.9%) | 685 (6.2%) |  |
| Personal service workers | 1,537 (7.4%) | 135 (1.8%) | 1,402 (13.0%) |  |
| Skilled blue collar workers | 3,116 (15.5%) | 2,665 (27.0%) | 451 (4.0%) |  |
| Unskilled blue collar workers | 1,239 (6.4%) | 780 (8.3%) | 459 (4.6%) |  |
| Agricultural workers | 201 (1.1%) | 142 (1.6%) | 59 (0.5%) |  |
| **Occupation (25 groups)** |  |  |  | <0.001 |
| Professionals working partially as self-employed | 93 (0.3%) | 26 (0.1%) | 67 (0.4%) |  |
| Public service professionals | 810 (2.2%) | 395 (2.3%) | 415 (2.1%) |  |
| Teaching and science professionals | 1,325 (4.2%) | 606 (4.0%) | 719 (4.5%) |  |
| Information and cultural professionals | 199 (1.6%) | 89 (1.8%) | 110 (1.3%) |  |
| Business and administration professionals | 1,080 (6.3%) | 574 (6.8%) | 506 (5.8%) |  |
| Engineering professionals | 897 (5.3%) | 715 (8.8%) | 182 (1.9%) |  |
| Primary, secondary, and vocational education teachers | 1,080 (3.5%) | 311 (2.4%) | 769 (4.5%) |  |
| Health and social work associate professionals | 2,451 (5.4%) | 375 (2.0%) | 2,076 (8.8%) |  |
| Clergy | 8 (0.0%) | 6 (0.0%) | 2 (0.0%) |  |
| Public service associate professionals | 980 (1.9%) | 297 (1.3%) | 683 (2.5%) |  |
| Business and administration associate professionals | 1,592 (8.0%) | 615 (6.3%) | 977 (9.6%) |  |
| Technicians | 1,144 (5.1%) | 944 (8.9%) | 200 (1.4%) |  |
| Foremen | 570 (2.6%) | 497 (4.5%) | 73 (0.7%) |  |
| Public service clerks and personal care workers | 4,329 (10.0%) | 811 (4.3%) | 3,518 (15.7%) |  |
| Protective services workers | 642 (2.2%) | 555 (4.0%) | 87 (0.5%) |  |
| Clerks | 1,426 (6.9%) | 175 (1.7%) | 1,251 (12.1%) |  |
| Sales workers | 880 (4.1%) | 195 (1.9%) | 685 (6.2%) |  |
| Personal service workers | 1,537 (7.4%) | 135 (1.8%) | 1,402 (13.0%) |  |
| Skilled industrial workers | 1,007 (5.3%) | 812 (8.6%) | 195 (2.0%) |  |
| Skilled craft workers | 1,240 (5.5%) | 1,069 (9.9%) | 171 (1.2%) |  |
| Drivers | 507 (2.6%) | 471 (4.9%) | 36 (0.4%) |  |
| Skilled handling, storage and transport workers | 362 (2.0%) | 313 (3.6%) | 49 (0.4%) |  |
| Unskilled industrial workers | 699 (3.7%) | 449 (5.0%) | 250 (2.5%) |  |
| Unskilled craft workers | 539 (2.7%) | 331 (3.2%) | 208 (2.2%) |  |
| Agricultural workers | 201 (1.1%) | 142 (1.6%) | 59 (0.5%) |  |
| **Economic activity (4 groups)** |  |  |  | <0.001 |
| Agriculture | 238 (1.1%) | 160 (1.6%) | 78 (0.6%) |  |
| Manufacturing | 3,120 (15.9%) | 2,168 (23.0%) | 952 (8.8%) |  |
| Construction | 1,086 (6.1%) | 944 (10.8%) | 142 (1.5%) |  |
| Services | 21,058 (76.9%) | 7,588 (64.6%) | 13,470 (89.1%) |  |
| **Economic activity (17 groups)** |  |  |  | <0.001 |
| Agriculture, forestry and fishing | 238 (1.1%) | 160 (1.6%) | 78 (0.6%) |  |
| Manufacture of food products, beverages, and tobacco products | 558 (2.5%) | 311 (3.0%) | 247 (2.0%) |  |
| Manufacture of coke and refined petroleum products | 27 (0.3%) | 23 (0.5%) | 4 (0.0%) |  |
| Manufacture of electrical, electronic and computer products, and machinery | 264 (2.1%) | 188 (3.1%) | 76 (1.1%) |  |
| Manufacture of transport equipment | 406 (1.9%) | 315 (2.9%) | 91 (0.9%) |  |
| Manufacture of other industrial products | 1,460 (7.0%) | 1,007 (10.0%) | 453 (4.1%) |  |
| Mining and quarrying, energy and water supply, waste management and remediation activities | 405 (2.0%) | 324 (3.4%) | 81 (0.7%) |  |
| Construction | 1,086 (6.1%) | 944 (10.8%) | 142 (1.5%) |  |
| Wholesale and retail trade, and repair of motor vehicles and motorcycles | 2,102 (11.7%) | 1,009 (12.1%) | 1,093 (11.3%) |  |
| Transportation and storage | 1,052 (5.9%) | 709 (8.2%) | 343 (3.6%) |  |
| Accommodation and food service activities | 520 (2.9%) | 215 (2.8%) | 305 (3.0%) |  |
| Information and communication | 514 (3.0%) | 332 (3.9%) | 182 (2.1%) |  |
| Financial and insurance activities | 671 (3.9%) | 253 (3.2%) | 418 (4.6%) |  |
| Real estate activities | 200 (1.1%) | 91 (1.0%) | 109 (1.3%) |  |
| Scientific and technical activities, and administrative and support service activities | 1,440 (8.7%) | 688 (8.8%) | 752 (8.7%) |  |
| Public administration, education, human health and social work activities | 12,891 (32.7%) | 3,896 (21.6%) | 8,995 (43.6%) |  |
| Other service activities | 1,668 (7.1%) | 395 (3.2%) | 1,273 (10.9%) |  |
| **Economic activity (38 groups)** |  |  |  | <0.001 |
| Agriculture, forestry and fishing | 238 (1.1%) | 160 (1.6%) | 78 (0.6%) |  |
| Mining and quarrying | 30 (0.2%) | 27 (0.3%) | 3 (0.0%) |  |
| Manufacture of food products, beverages, and tobacco products | 558 (2.5%) | 311 (3.0%) | 247 (2.0%) |  |
| Manufacture of textiles, wearing apparel, leather products, and footwear | 112 (0.4%) | 40 (0.3%) | 72 (0.5%) |  |
| Manufacture of wood and paper, and printing | 165 (0.7%) | 111 (1.0%) | 54 (0.4%) |  |
| Manufacture of coke and refined petroleum products | 27 (0.3%) | 23 (0.5%) | 4 (0.0%) |  |
| Manufacture of chemicals and chemical products | 153 (0.8%) | 103 (1.1%) | 50 (0.4%) |  |
| Manufacture of basic pharmaceutical products and pharmaceutical preparations | 126 (0.7%) | 55 (0.7%) | 71 (0.8%) |  |
| Manufacture of rubber and plastic products, and other non-metallic mineral products. | 264 (1.3%) | 191 (1.9%) | 73 (0.7%) |  |
| Manufacture of basic metals and fabricated metal products, except machinery and equipment | 427 (2.1%) | 348 (3.4%) | 79 (0.7%) |  |
| Manufacture of computer, electronic, and optical products | 79 (0.6%) | 47 (0.9%) | 32 (0.4%) |  |
| Manufacture of electrical equipment | 45 (0.3%) | 24 (0.3%) | 21 (0.3%) |  |
| Manufacture of machinery and equipment n.e.c. | 140 (1.2%) | 117 (1.9%) | 23 (0.4%) |  |
| Manufacture of transport equipement | 406 (1.9%) | 315 (2.9%) | 91 (0.9%) |  |
| Other manufacturing activities, and repair and installation of machinery and equipment | 213 (1.1%) | 159 (1.6%) | 54 (0.6%) |  |
| Electricity, gas, steam and air conditioning supply | 185 (1.2%) | 146 (2.0%) | 39 (0.4%) |  |
| Water supply, sewerage, waste management and remediation activities | 190 (0.7%) | 151 (1.1%) | 39 (0.3%) |  |
| Construction | 1,086 (6.1%) | 944 (10.8%) | 142 (1.5%) |  |
| Wholesale and retail trade, and repair of motor vehicles and motorcycles | 2,102 (11.7%) | 1,009 (12.1%) | 1,093 (11.3%) |  |
| Transportation and storage | 1,052 (5.9%) | 709 (8.2%) | 343 (3.6%) |  |
| Accommodation and food service activities | 520 (2.9%) | 215 (2.8%) | 305 (3.0%) |  |
| Publishing, programming and broadcasting activities | 147 (1.3%) | 83 (1.5%) | 64 (1.0%) |  |
| Telecommunications | 135 (0.5%) | 87 (0.7%) | 48 (0.4%) |  |
| Computer programming, consultancy and related activities, and information service activities | 232 (1.2%) | 162 (1.7%) | 70 (0.7%) |  |
| Financial and insurance activities | 671 (3.9%) | 253 (3.2%) | 418 (4.6%) |  |
| Real estate activities | 200 (1.1%) | 91 (1.0%) | 109 (1.3%) |  |
| Legal and accounting, management consultancy, architectural and engineering, and technical testing and analysis activities | 420 (3.0%) | 177 (2.6%) | 243 (3.4%) |  |
| Scientific research and development | 220 (1.0%) | 121 (1.1%) | 99 (1.0%) |  |
| Other scientific and technical activities | 175 (1.1%) | 84 (1.1%) | 91 (1.2%) |  |
| Administrative and support service activities | 625 (3.5%) | 306 (3.9%) | 319 (3.2%) |  |
| Public administration | 3,873 (9.7%) | 1,867 (9.9%) | 2,006 (9.5%) |  |
| Education | 2,981 (9.1%) | 894 (6.1%) | 2,087 (12.0%) |  |
| Human health activities | 4,543 (8.0%) | 903 (3.6%) | 3,640 (12.4%) |  |
| Residential care activities, and social work activities without accommodation | 1,494 (5.8%) | 232 (1.9%) | 1,262 (9.8%) |  |
| Arts, entertainment, and recreation activities | 331 (1.3%) | 142 (1.4%) | 189 (1.3%) |  |
| Other service activities | 446 (1.5%) | 134 (0.9%) | 312 (2.0%) |  |
| Activities of households | 851 (4.2%) | 100 (0.8%) | 751 (7.5%) |  |
| Activities of extraterritorial organisations and bodies | 40 (0.1%) | 19 (0.1%) | 21 (0.1%) |  |
| **Public/private sector** |  |  |  | <0.001 |
| Public | 11,256 (25.1%) | 3,927 (19.9%) | 7,329 (30.3%) |  |
| Private | 14,380 (74.9%) | 6,999 (80.1%) | 7,381 (69.7%) |  |
| **Company size** |  |  |  | <0.001 |
| 1-49 | 4,847 (26.1%) | 2,083 (23.5%) | 2,764 (28.7%) |  |
| 50-499 | 3,174 (14.9%) | 1,563 (16.5%) | 1,611 (13.3%) |  |
| 500 or more | 16,638 (59.0%) | 6,845 (60.0%) | 9,793 (58.0%) |  |
| **Permanent/temporary work contract** |  |  |  | 0.038 |
| Permanent | 23,732 (91.5%) | 10,233 (92.2%) | 13,499 (90.8%) |  |
| Temporary | 1,890 (8.5%) | 691 (7.8%) | 1,199 (9.2%) |  |
| **Full/part time work** |  |  |  | <0.001 |
| Full time | 20,753 (82.2%) | 10,371 (95.6%) | 10,382 (69.0%) |  |
| Part time | 4,691 (17.8%) | 503 (4.4%) | 4,188 (31.0%) |  |

n (w%): unweighted number (weighted %)

P-value for the comparison between genders (Rao-Scott Chi-2 test)

Supplementary Table S2. Age and employment variables in association with workplace bullying among the study sample, and among men and women separately: results from robust Poisson regression models

|  | All  (N=24,338) | | | Men  (N=10,378) | | | Women  (N=13,960) | | |
| --- | --- | --- | --- | --- | --- | --- | --- | --- | --- |
|  | PR | 95% CI | P-value | PR | 95% CI | P-value | PR | 95% CI | P-value |
| **Gender** |  |  | <0.001 |  |  |  |  |  |  |
| Men | 1 |  |  |  |  |  |  |  |  |
| Women | **1.14***** | **1.06; 1.23** |  |  |  |  |  |  |  |
| **Age (years)** |  |  | <0.001 |  |  | <0.001 |  |  | <0.001 |
| <30 | **1.41***** | **1.25; 1.59** |  | **1.60***** | **1.35; 1.89** |  | **1.24*** | **1.05; 1.47** |  |
| [30-40[ | **1.37***** | **1.25; 1.51** |  | **1.44***** | **1.26; 1.65** |  | **1.31***** | **1.16; 1.49** |  |
| [40-50[ | **1.18***** | **1.08; 1.30** |  | **1.36***** | **1.19; 1.55** |  | 1.06 | 0.93; 1.19 |  |
| >=50 | 1 |  |  | 1 |  |  | 1 |  |  |
| **Occupation (4 groups)** |  |  | 0.039 |  |  | 0.229 |  |  | 0.004 |
| Professionals/managers | 1.05 | 0.94; 1.17 |  | 0.86 | 0.73; 1.01 |  | **1.19*** | **1.04; 1.36** |  |
| Associate professionals/technicians | **1.14**** | **1.04; 1.24** |  | 0.97 | 0.83; 1.13 |  | **1.22***** | **1.09; 1.36** |  |
| Clerks/service workers | 1 |  |  | 1 |  |  | 1 |  |  |
| Blue collar workers | 1.10 | 0.99; 1.24 |  | 0.97 | 0.83; 1.14 |  | 1.13 | 0.94; 1.36 |  |
| **Economic activity (4 groups)** |  |  | 0.039 |  |  | 0.069 |  |  | 0.399 |
| Agriculture | **0.62*** | **0.43; 0.91** |  | **0.62*** | **0.40; 0.97** |  | 0.65 | 0.30; 1.37 |  |
| Manufacturing | 1.04 | 0.94; 1.16 |  | 1.02 | 0.89; 1.16 |  | 1.09 | 0.92; 1.28 |  |
| Construction | 0.88 | 0.71; 1.09 |  | 0.83 | 0.67; 1.03 |  | 1.23 | 0.69; 2.20 |  |
| Services | 1 |  |  | 1 |  |  | 1 |  |  |
| **Public/private sector** |  |  | 0.218 |  |  | 0.916 |  |  | 0.202 |
| Public | 1.05 | 0.97; 1.15 |  | 1.01 | 0.89; 1.14 |  | 1.08 | 0.96; 1.21 |  |
| Private | 1 |  |  | 1 |  |  | 1 |  |  |
| **Company size** |  |  | <0.001 |  |  | <0.001 |  |  | 0.012 |
| 1-49 | 1 |  |  | 1 |  |  | 1 |  |  |
| 50-499 | **1.31***** | **1.15; 1.48** |  | **1.38***** | **1.16; 1.64** |  | **1.25*** | **1.05; 1.49** |  |
| 500 or more | **1.35***** | **1.20; 1.52** |  | **1.44***** | **1.22; 1.69** |  | **1.27**** | **1.07; 1.49** |  |
| **Permanent/temporary work contract** |  |  | 0.836 |  |  | 0.764 |  |  | 0.998 |
| Permanent | 1 |  |  | 1 |  |  | 1 |  |  |
| Temporary | 0.99 | 0.86; 1.13 |  | 0.97 | 0.78; 1.20 |  | 1.00 | 0.84; 1.20 |  |
| **Part/full time work** |  |  | 0.044 |  |  | 0.855 |  |  | 0.039 |
| Full time | 1 |  |  | 1 |  |  | 1 |  |  |
| Part time | **0.90*** | **0.82; 1.00** |  | 0.98 | 0.79; 1.22 |  | **0.89*** | **0.80; 0.99** |  |

Poisson regression models with robust variance estimation using weighted data

PR: prevalence ratio, CI: confidence interval

All variables were included simultaneously in the models

Reference group for occupation and economic activity was chosen among the groups with the highest sample size for both men and women

* p<0.05, **p<0.01, ***<0.001

Supplementary Table S3. Age and employment variables in association with workplace bullying among the study sample, and among men and women separately: results from robust Poisson regression models

|  | All  (N=24,332) | | | Men  (N=10,370) | | | Women  (N=13,952) | | |
| --- | --- | --- | --- | --- | --- | --- | --- | --- | --- |
|  | PR | 95% CI | P-value | PR | 95% CI | P-value | PR | 95% CI | P-value |
| **Gender** |  |  | 0.002 |  |  |  |  |  |  |
| Men | 1 |  |  |  |  |  |  |  |  |
| Women | **1.13**** | **1.05; 1.22** |  |  |  |  |  |  |  |
| **Age (years)** |  |  | <0.001 |  |  | <0.001 |  |  | <0.001 |
| <30 | **1.36***** | **1.20; 1.53** |  | **1.57***** | **1.33; 1.86** |  | **1.20*** | **1.02; 1.41** |  |
| [30-40[ | **1.35***** | **1.23; 1.47** |  | **1.43***** | **1.25; 1.63** |  | **1.29***** | **1.14; 1.46** |  |
| [40-50[ | **1.17***** | **1.07; 1.28** |  | **1.35***** | **1.18; 1.54** |  | 1.04 | 0.93; 1.17 |  |
| >=50 | 1 |  |  | 1 |  |  | 1 |  |  |
| **Occupation (14 groups)** |  |  | <0.001 |  |  | 0.056 |  |  | <0.001 |
| Professionals working partially as self-employed | 0.79 | 0.49; 1.26 |  | 1.50 | 0.80; 2.79 |  | 0.60 | 0.34; 1.05 |  |
| Public service; teaching; science; and cultural professionals | 1.07 | 0.94; 1.22 |  | 1.00 | 0.81; 1.23 |  | 1.15 | 0.98; 1.34 |  |
| Business; administration; and engineering professionals | 0.84 | 0.70; 1.00 |  | 0.82 | 0.64; 1.05 |  | 0.88 | 0.68; 1.15 |  |
| Teaching; health; and public service associate professionals | 1.07 | 0.97; 1.19 |  | 1.18 | 0.97; 1.43 |  | 1.05 | 0.93; 1.18 |  |
| Business and administration associate professionals | 0.99 | 0.83; 1.19 |  | 0.98 | 0.75; 1.27 |  | 1.00 | 0.80; 1.27 |  |
| Technicians | 0.91 | 0.75; 1.09 |  | 0.89 | 0.70; 1.14 |  | 1.08 | 0.80; 1.45 |  |
| Foremen | 0.92 | 0.72; 1.17 |  | 0.93 | 0.70; 1.24 |  | 0.99 | 0.58; 1.69 |  |
| Public service clerks and workers | 1 |  |  | 1 |  |  | 1 |  |  |
| Clerks | 0.97 | 0.80; 1.17 |  | 1.28 | 0.91; 1.80 |  | 0.89 | 0.71; 1.12 |  |
| Sales workers | 0.97 | 0.77; 1.22 |  | 1.19 | 0.79; 1.78 |  | 0.92 | 0.69; 1.22 |  |
| Personal service workers | **0.50***** | **0.39; 0.63** |  | 0.82 | 0.49; 1.36 |  | **0.45***** | **0.34; 0.60** |  |
| Skilled blue collar workers | 0.95 | 0.82; 1.11 |  | 0.99 | 0.81; 1.21 |  | 0.96 | 0.73; 1.25 |  |
| Unskilled blue collar workers | 1.01 | 0.85; 1.22 |  | 1.07 | 0.84; 1.37 |  | 0.98 | 0.73; 1.30 |  |
| Agricultural workers | **0.41**** | **0.22; 0.73** |  | **0.49*** | **0.24; 0.98** |  | **0.25*** | **0.08; 0.80** |  |
| **Economic activity (17 groups)** |  |  | 0.028 |  |  | 0.115 |  |  | <0.001 |
| Agriculture; forestry and fishing | 0.99 | 0.62; 1.59 |  | 0.99 | 0.58; 1.69 |  | 0.94 | 0.40; 2.24 |  |
| Manufacture of food products; beverages; and tobacco products | 1.00 | 0.80; 1.26 |  | 0.95 | 0.69; 1.30 |  | 1.02 | 0.74; 1.41 |  |
| Manufacture of coke and refined petroleum products | **1.91*** | **1.08; 3.38** |  | 1.64 | 0.72; 3.73 |  | **2.75***** | **1.97; 3.83** |  |
| Manufacture of electrical; electronic and computer products; and machinery | 1.14 | 0.87; 1.50 |  | 1.02 | 0.73; 1.44 |  | 1.31 | 0.82; 2.10 |  |
| Manufacture of transport equipment | 0.84 | 0.65; 1.10 |  | 0.78 | 0.57; 1.06 |  | 0.92 | 0.56; 1.51 |  |
| Manufacture of other industrial products | 1.10 | 0.92; 1.30 |  | 1.06 | 0.84; 1.33 |  | 1.03 | 0.80; 1.34 |  |
| Mining and quarrying; energy and water supply; waste management and remediation activities | 1.03 | 0.80; 1.33 |  | 0.91 | 0.66; 1.25 |  | 1.32 | 0.90; 1.92 |  |
| Construction | 0.87 | 0.69; 1.10 |  | 0.80 | 0.61; 1.04 |  | 1.15 | 0.65; 2.05 |  |
| Wholesale and retail trade; and repair of motor vehicles and motorcycles | 0.97 | 0.82; 1.16 |  | 0.92 | 0.72; 1.18 |  | 0.99 | 0.78; 1.24 |  |
| Transportation and storage | 0.91 | 0.77; 1.09 |  | 0.84 | 0.66; 1.07 |  | 0.97 | 0.75; 1.27 |  |
| Accommodation and food service activities | **1.40**** | **1.12; 1.75** |  | 1.34 | 0.96; 1.88 |  | 1.28 | 0.94; 1.75 |  |
| Information and communication | 0.95 | 0.73; 1.26 |  | 0.96 | 0.66; 1.41 |  | 0.93 | 0.65; 1.33 |  |
| Financial and insurance activities | 1.01 | 0.78; 1.29 |  | 0.76 | 0.50; 1.14 |  | 1.17 | 0.86; 1.61 |  |
| Real estate activities | 1.08 | 0.65; 1.81 |  | 0.49 | 0.20; 1.16 |  | 1.37 | 0.80; 2.35 |  |
| Scientific and technical activities; and administrative and support service activities | 1.10 | 0.94; 1.29 |  | 1.08 | 0.86; 1.36 |  | 1.06 | 0.85; 1.32 |  |
| Public administration; education; human health and social work activities | 1 |  |  | 1 |  |  | 1 |  |  |
| Other service activities | 0.93 | 0.77; 1.13 |  | 0.97 | 0.72; 1.31 |  | 0.90 | 0.71; 1.15 |  |
| **Public/private sector** |  |  | 0.416 |  |  | 0.229 |  |  | 0.990 |
| Public | 0.95 | 0.83; 1.08 |  | 0.89 | 0.73; 1.08 |  | 1.00 | 0.84; 1.19 |  |
| Private | 1 |  |  | 1 |  |  | 1 |  |  |
| **Company size** |  |  | <0.001 |  |  | <0.001 |  |  | 0.359 |
| 1-49 | 1 |  |  | 1 |  |  | 1 |  |  |
| 50-499 | **1.23**** | **1.08; 1.40** |  | **1.39***** | **1.16; 1.66** |  | 1.11 | 0.93; 1.33 |  |
| 500 or more | **1.30***** | **1.15; 1.47** |  | **1.50***** | **1.27; 1.78** |  | 1.12 | 0.95; 1.32 |  |
| **Permanent/temporary work contract** |  |  | 0.937 |  |  | 0.629 |  |  | 0.709 |
| Permanent | 1 |  |  | 1 |  |  | 1 |  |  |
| Temporary | 1.01 | 0.88; 1.15 |  | 0.95 | 0.76; 1.18 |  | 1.03 | 0.87; 1.23 |  |
| **Part/full time work** |  |  | 0.115 |  |  | 0.534 |  |  | 0.118 |
| Full time | 1 |  |  | 1 |  |  | 1 |  |  |
| Part time | 0.93 | 0.84; 1.02 |  | 0.93 | 0.75; 1.16 |  | 0.92 | 0.83; 1.02 |  |

Poisson regression models with robust variance estimation using weighted data

PR: prevalence ratio, CI: confidence interval

All variables were included simultaneously in the models

Reference group for occupation and economic activity was chosen among the groups with the highest sample size for both men and women

* p<0.05, **p<0.01, ***<0.001

Supplementary Table S4. Age and employment variables in association with workplace bullying among the study sample, and among men and women separately: results of forward stepwise robust Poisson regression models

|  | All  (N=24,350) | | | Men  (N=10,491) | | | Women  (N=14,028) | | |
| --- | --- | --- | --- | --- | --- | --- | --- | --- | --- |
|  | PR | 95% CI | P-value | PR | 95% CI | P-value | PR | 95% CI | P-value |
| **Gender†** |  |  | <0.001 |  |  |  |  |  |  |
| Men | 1 |  |  |  |  |  |  |  |  |
| Women | **1.15***** | **1.06; 1.24** |  |  |  |  |  |  |  |
| **Age (years)** |  |  | <0.001 |  |  | <0.001 |  |  | <0.001 |
| <30 | **1.40***** | **1.24; 1.57** |  | **1.60***** | **1.36; 1.88** |  | **1.22*** | **1.03; 1.44** |  |
| [30-40[ | **1.37***** | **1.25; 1.50** |  | **1.44***** | **1.26; 1.65** |  | **1.29***** | **1.14; 1.46** |  |
| [40-50[ | **1.18***** | **1.08; 1.30** |  | **1.36***** | **1.19; 1.56** |  | 1.04 | 0.92; 1.18 |  |
| >=50 | 1 |  |  | 1 |  |  | 1 |  |  |
| **Company size** |  |  | <0.001 |  |  | <0.001 |  |  | 0.002 |
| 1-49 | 1 |  |  | 1 |  |  | 1 |  |  |
| 50-499 | **1.31***** | **1.15; 1.48** |  | **1.40***** | **1.18; 1.67** |  | **1.24*** | **1.04; 1.48** |  |
| 500 or more | **1.38***** | **1.24; 1.53** |  | **1.50***** | **1.31; 1.72** |  | **1.30***** | **1.13; 1.50** |  |
| **Full/part time work** |  |  | 0.040 |  |  |  |  |  | 0.026 |
| Full time | 1 |  |  |  |  |  | 1 |  |  |
| Part time | **0.90*** | **0.82; 1.00** |  |  |  |  | **0.89*** | **0.80; 0.99** |  |
| **Occupation (4 groups)** |  |  | 0.038 |  |  |  |  |  | 0.005 |
| Professionals/managers | 1.05 | 0.94; 1.17 |  |  |  |  | **1.18*** | **1.03; 1.35** |  |
| Associate professionals/technicians | **1.14**** | **1.04; 1.24** |  |  |  |  | **1.21***** | **1.09; 1.36** |  |
| Clerks/service workers | 1 |  |  |  |  |  | 1 |  |  |
| Blue collar workers | 1.10 | 0.98; 1.23 |  |  |  |  | 1.12 | 0.95; 1.33 |  |
| **Economic activity (4 groups)** |  |  | 0.045 |  |  |  |  |  |  |
| Agriculture | **0.62*** | **0.42; 0.91** |  |  |  |  |  |  |  |
| Manufacturing | 1.03 | 0.93; 1.13 |  |  |  |  |  |  |  |
| Construction | 0.87 | 0.71; 1.08 |  |  |  |  |  |  |  |
| Services | 1 |  |  |  |  |  |  |  |  |

Poisson regression models with robust variance estimation using weighted data

PR: prevalence ratio, CI: confidence interval

† Forced variable in the model

Variables presented in the order of selection for all and for men

The order of selection for women was: occupation, age, company size, and full/part time work

* p<0.05, **p<0.01, ***<0.001

Supplementary Table S5. Age and employment variables in association with workplace bullying among the study sample, and among men and women separately: results of forward stepwise robust Poisson regression models

|  | All  (N=25,517) | | | Men  (N=10,491) | | | Women  (N=14,626) | | |
| --- | --- | --- | --- | --- | --- | --- | --- | --- | --- |
|  | PR | 95% CI | P-value | PR | 95% CI | P-value | PR | 95% CI | P-value |
| **Gender†** |  |  | 0.006 |  |  |  |  |  |  |
| Men | 1 |  |  |  |  |  |  |  |  |
| Women | **1.11**** | **1.03; 1.20** |  |  |  |  |  |  |  |
| **Occupation (14 groups)** |  |  | <0.001 |  |  |  |  |  | <0.001 |
| Professionals working partially as self-employed | 0.78 | 0.49; 1.25 |  |  |  |  | **0.57*** | **0.32; 0.99** |  |
| Public service, teaching, science, and cultural professionals | 1.07 | 0.94; 1.21 |  |  |  |  | **1.18*** | **1.01; 1.37** |  |
| Business, administration, and engineering professionals | 0.86 | 0.72; 1.02 |  |  |  |  | 0.89 | 0.69; 1.14 |  |
| Teaching, health, and public service associate professionals | 1.07 | 0.97; 1.19 |  |  |  |  | 1.03 | 0.92; 1.16 |  |
| Business and administration associate professionals | 1.02 | 0.86; 1.20 |  |  |  |  | 1.01 | 0.82; 1.24 |  |
| Technicians | 0.92 | 0.77; 1.11 |  |  |  |  | 1.12 | 0.85; 1.50 |  |
| Foremen | 0.93 | 0.74; 1.18 |  |  |  |  | 0.96 | 0.57; 1.61 |  |
| Public service clerks and workers | 1 |  |  |  |  |  | 1 |  |  |
| Clerks | 0.98 | 0.82; 1.18 |  |  |  |  | 0.88 | 0.72; 1.07 |  |
| Sales workers | 0.98 | 0.78; 1.22 |  |  |  |  | 0.95 | 0.73; 1.24 |  |
| Personal service workers | **0.49***** | **0.39; 0.62** |  |  |  |  | **0.42***** | **0.32; 0.54** |  |
| Skilled blue collar workers | 0.97 | 0.83; 1.12 |  |  |  |  | 0.90 | 0.70; 1.16 |  |
| Unskilled blue collar workers | 1.03 | 0.86; 1.23 |  |  |  |  | 0.90 | 0.70; 1.17 |  |
| Agricultural workers | **0.52*** | **0.30; 0.90** |  |  |  |  | **0.30*** | **0.11; 0.83** |  |
| **Age (years)** |  |  | <0.001 |  |  | <0.001 |  |  | <0.001 |
| <30 | **1.37***** | **1.22; 1.53** |  | **1.60***** | **1.36; 1.88** |  | **1.21*** | **1.04; 1.41** |  |
| [30-40[ | **1.35***** | **1.23; 1.48** |  | **1.44***** | **1.26; 1.65** |  | **1.29***** | **1.15; 1.46** |  |
| [40-50[ | **1.17***** | **1.07; 1.28** |  | **1.36***** | **1.19; 1.56** |  | 1.05 | 0.94; 1.18 |  |
| >=50 | 1 |  |  | 1 |  |  | 1 |  |  |
| **Company size** |  |  | <0.001 |  |  | <0.001 |  |  |  |
| 1-49 | 1 |  |  | 1 |  |  |  |  |  |
| 50-499 | **1.24***** | **1.09; 1.41** |  | **1.40***** | **1.18; 1.67** |  |  |  |  |
| 500 or more | **1.30***** | **1.16; 1.45** |  | **1.50***** | **1.31; 1.72** |  |  |  |  |
| **Economic activity (17 groups)** |  |  | 0.020 |  |  |  |  |  | <0.001 |
| Agriculture, forestry and fishing | 1.07 | 0.69; 1.67 |  |  |  |  | 0.90 | 0.40; 2.02 |  |
| Manufacture of food products, beverages, and tobacco products | 1.04 | 0.83; 1.29 |  |  |  |  | 0.99 | 0.73; 1.36 |  |
| Manufacture of coke and refined petroleum products | **1.97*** | **1.12; 3.48** |  |  |  |  | **2.74***** | **1.93; 3.89** |  |
| Manufacture of electrical, electronic and computer products, and machinery | 1.17 | 0.90; 1.53 |  |  |  |  | 1.37 | 0.86; 2.17 |  |
| Manufacture of transport equipment | 0.87 | 0.68; 1.12 |  |  |  |  | 0.93 | 0.57; 1.51 |  |
| Manufacture of other industrial products | 1.13 | 0.96; 1.32 |  |  |  |  | 1.04 | 0.81; 1.32 |  |
| Mining and quarrying, energy and water supply, waste management and remediation activities | 1.05 | 0.82; 1.35 |  |  |  |  | 1.34 | 0.94; 1.92 |  |
| Construction | 0.89 | 0.71; 1.12 |  |  |  |  | 1.15 | 0.68; 1.94 |  |
| Wholesale and retail trade, and repair of motor vehicles and motorcycles | 1.00 | 0.85; 1.18 |  |  |  |  | 0.95 | 0.77; 1.18 |  |
| Transportation and storage | 0.94 | 0.79; 1.10 |  |  |  |  | 1.04 | 0.81; 1.33 |  |
| Accommodation and food service activities | **1.43**** | **1.15; 1.78** |  |  |  |  | 1.24 | 0.92; 1.67 |  |
| Information and communication | 0.97 | 0.73; 1.28 |  |  |  |  | 0.91 | 0.65; 1.28 |  |
| Financial and insurance activities | 1.03 | 0.81; 1.32 |  |  |  |  | 1.19 | 0.87; 1.61 |  |
| Real estate activities | 1.11 | 0.66; 1.86 |  |  |  |  | 1.26 | 0.73; 2.18 |  |
| Scientific and technical activities, and administrative and support service activities | 1.12 | 0.96; 1.30 |  |  |  |  | 1.02 | 0.82; 1.25 |  |
| Public administration, education, human health and social work activities | 1 |  |  |  |  |  | 1 |  |  |
| Other service activities | 0.92 | 0.76; 1.11 |  |  |  |  | 0.85 | 0.67; 1.07 |  |

Poisson regression models with robust variance estimation using weighted data

PR: prevalence ratio, CI: confidence interval

† Forced variable in the model

Variables presented in the order of selection for all and for men

The order of selection for women was: economic activity, occupation, and age

* p<0.05, **p<0.01, ***<0.001

Supplementary Figure S1. Associations of gender and employment variables with the 12-month prevalence of exposure to workplace bullying among the study sample (robust Poisson regression models without adjustment for age, each variable studied separately)


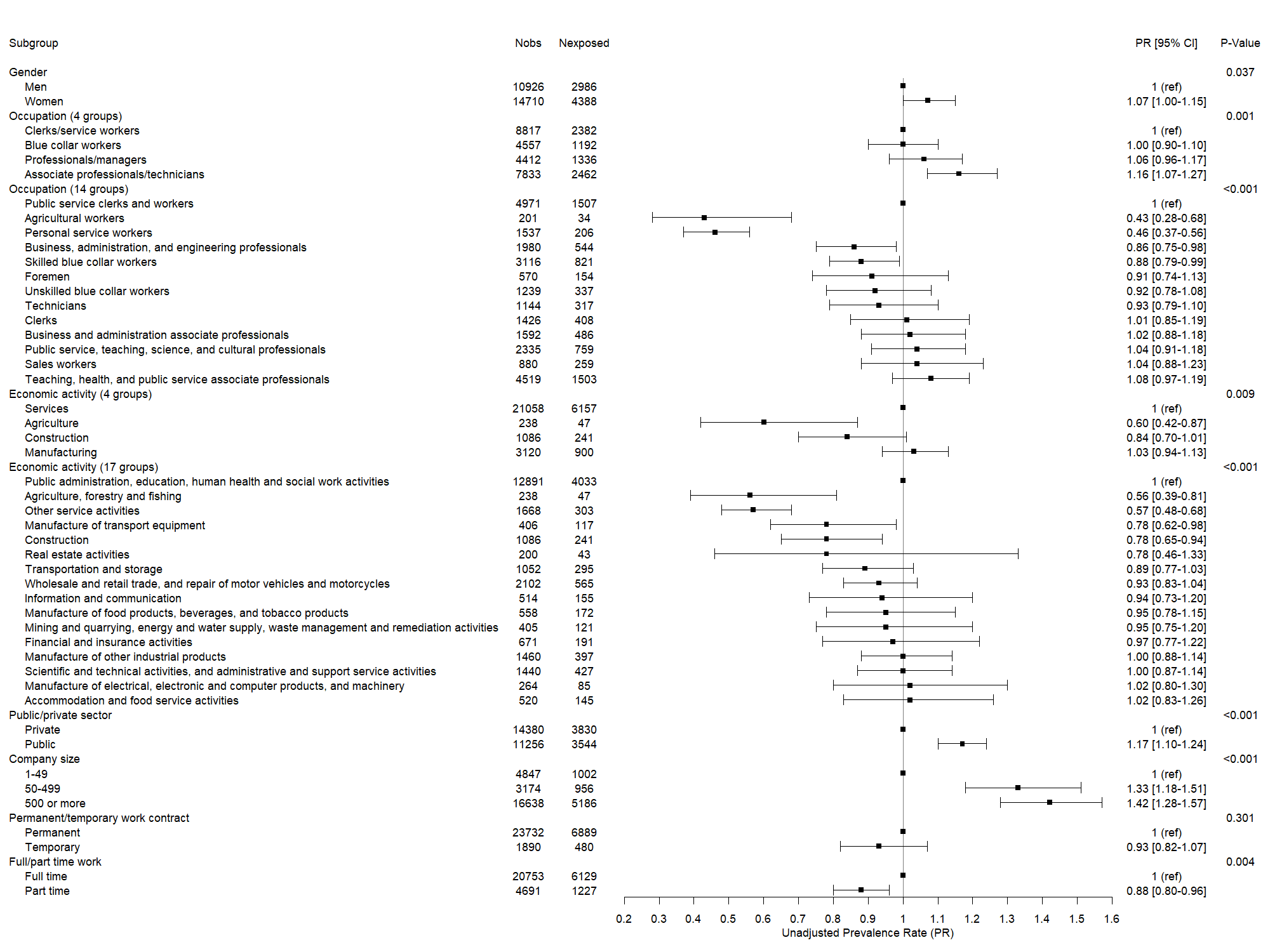


Supplementary Figure S2. Associations of gender and employment variables with the 12-month prevalence of exposure to workplace bullying among the study sample (robust Poisson regression models with adjustment for age, each variable studied separately)


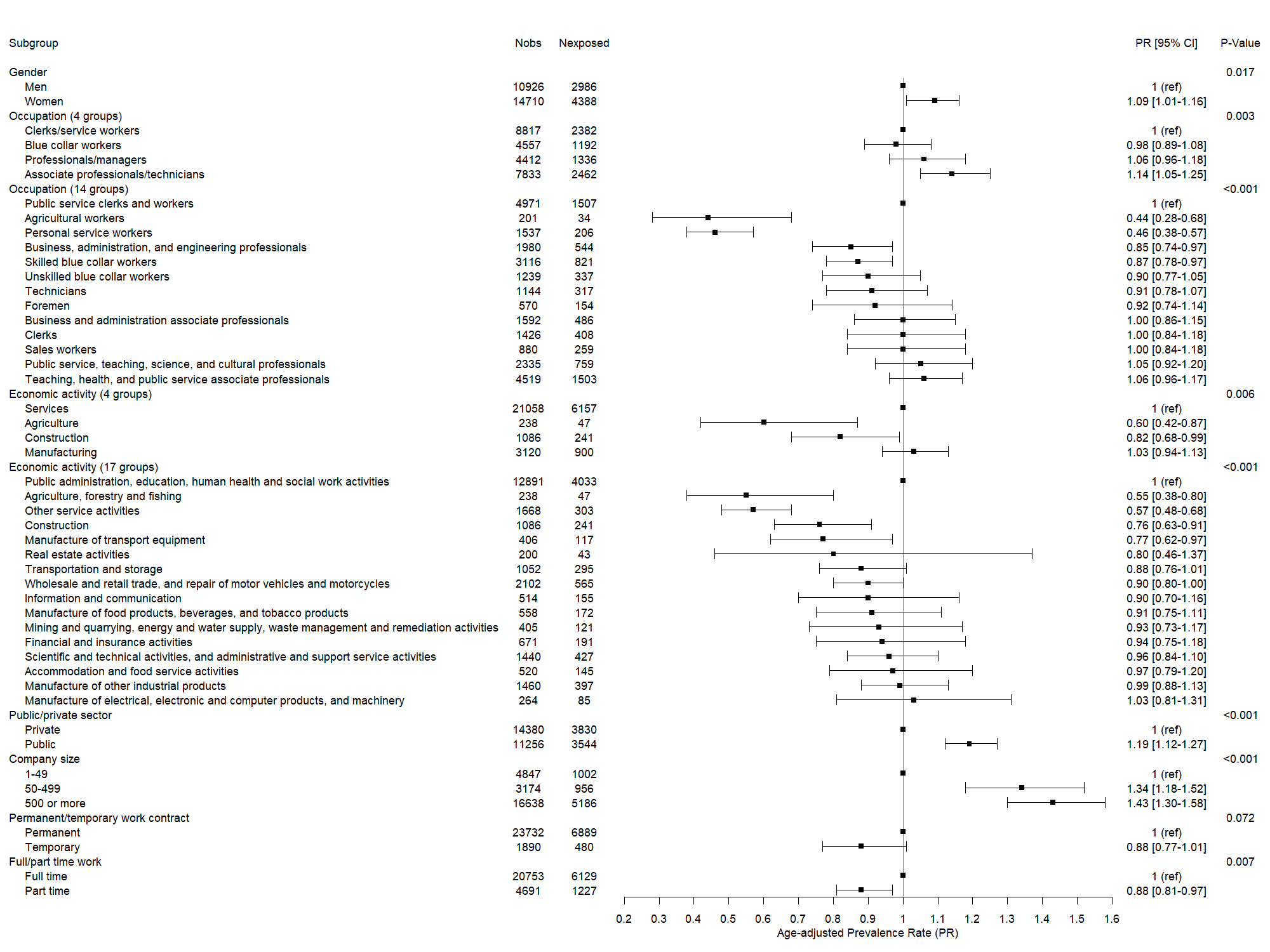

Supplement: Supplementary file 1 — Supplementary file1 (DOCX 236 KB) [file 420_2022_1916_MOESM1_ESM.docx]
